# Abstracts and Gleanings

**Published:** 1887-01-01

**Authors:** 


### ABSTRACTS AND GLEANINGS.

**Recent Progress in the Investigation of Hog Cholera.**—The interest which this Association (American Public Health Association, Toronto, Can.) was pleased to show in the remarks that I had the honor to make on the cause of hog cholera at the meeting of one year ago encourages me to give a brief account of some more recent investigations of the same disease.

The experiments of the year, which have been very numerous, abundantly confirm the conclusion that this disease is caused by a bacterium—that is, an organism which exists in the form of a short rod and is generally united in twos, and sometimes in fours. It is mobile, stains deeply around its entire periphery, and, so far as we have been able to ascertain, does not form spores. It grows on nutrient gelatine without liquefying it. The cultures of it are fatal to mice, rabbits, Guinea pigs, pigeons, and swine, but they do not seriously affect fowls. * * *

While this disease, as it occurs in the herds of the country, is extremely virulent and fatal, a considerable dose of the virus may be injected hypodermically without producing serious effects in swine. We have inoculated, in this way with $\frac{1}{4}$ cc. and have lost no animals from this dose. By increasing the quantity to $\frac{1}{2}$ cc. the operation becomes more dangerous, but even in this case only one animal out of eight succumbed Such inoculations evidently confer a certain degree of immunity, because the individuals which have been operated upon in this way may afterward be inoculated with 2, $2\frac{1}{2}$, or even 3 cc. without any serious results.

The test of inoculation, however, is certainly far from being as severe a test of immunity as actual exposure to the contagion in infected pens. Thus pigs which had received $\frac{3}{4}$ to $\frac{1}{2}$ cc. of virus for a first dose were not sufficiently protected to enable them to resist exposure to sick pigs in infected places.

If so large a dose of virus may be injected into the tissues without producing fatal effects, it seems difficult, at first, to explain how the contagion, carried on the feet of animals and people, and in many other ways in which but a small quantity can be transported, is able to infect individuals and start new outbreaks. Yet this is evidently true, or the plague could not spread from herd to herd as frequently as we know it does. It would seem that the small quantity of contagion which is carried and deposited upon the new premises must find there a nidus suitable for its multiplication before herds can be infected in that way.

To test this hypothesis, we have placed the organisms in hay infusion, and find that it is able to multiply in such a liquid. But what is more remarkable is its life and rapid development in ordinary water. An idea of this can be obtained from the following experiment: 10 cc. of Potomac water, such as is supplied to the families of Washington, was sterilized by heat and as many germs of this disease added as would be carried on a small loop of platinum wire dipped into a culture liquid. It was then tested to determine the number of microbes that had been added to each cc., and there were found to be 26240. This was September 8th. On September 9th there were 1,276,000 germs to the cc., showing that they had increased nearly eightfold in twenty-four hours. September 10th there were 1,296,000 germs to the cc., an increase of fiftyfold in forty-eight hours. September 13 there were 2,608,200, an increase of one hundred fold in five days. The limit of growth to each cc. of water is reached by the last figures given, and after this period the number of living germs begins to decrease. We have here, consequently, the demonstration that this pathogenic organism may multiply to a wonderful extent in water as pure as that of the ordinary running streams of the country. It is true that in this case the disease germ did not contend with the ordinary germs with which all waters in nature are more or less contaminated, and it is possible that their development would be somewhat affected by the presence of these other species. It may be admitted, however, that these germs have the power of living and multiplying in water or in moist organic matters such as usually exist in enclosures where hogs are kept.

Drying is destructive to this germ, though it retains its vitality for a considerable time. With drops of culture liquid dried on cover glasses we found that, in some cases, the germs would all be destroyed after eight or nine days, but in other cases there were still living germs after twenty-two days. In small pieces of spleen not larger than a pin-head, which were thoroughly dried, there were still living germs at the end of forty-nine days.

The tests of disinfectants have given some unexpected results, and emphasize the necessity of determining the effects of these agents upon each specific microbe. This organism is destroyed by a solution of permanganate of potash of 1:5000 acting for fifteen minutes. Of sulphuric acid, 1:2000 is required; of carbolic acid, 1:80; of mercuric chloride, 1:75000, and in some preliminary experiments with mercuric iodide this agent was found efficient in the strength of 1:1000000.—D. E. SALMON, D. V. M., *in Sanitarian.*

**Poisoning by Milk.**— We have been taught that milk may transmit the poisons of typhoid fever, of scarlatina, and other infectious diseases, to those drinking the same. Other poisons may be transmitted by the milk from one place or person to others far distant. It is even claimed that tuberculosis may be transmitted. But to this phase of milk poisoning we do not refer just now.

We desire to call attention to poisons developed in milk aside from any substance or poison which has entered it from without. Dr. Newton (*Medical News*, Sept. 25) reports some investigations of importance bearing upon this matter. Spoiled milk has been known to become poisonous under certain circumstances. Briefly, the story of Dr. Newton's researches is as follows:

On August 7, forty-three persons were taken sick at two hotels at Long Branch. The symptoms were the same in all the cases, viz.: those of gastro-intestinal irritation. A week after this series of cases occurred thirty persons were taken sick in the same manner at another hotel.

In searching for the cause of this outbreak it was found that every article of diet could be eliminated except milk. It was found that one dealer had supplied all the milk to these hotels. Farther, it appeared that only those were sick who partook of milk supplied at night. The cows, farms, food and drink of the cows, etc., were found to be entirely right. Finally it was found that the cows were milked at noon and at midnight. The noon milking alone produced the illness. This, while hot, in the heat of the day, was placed in cans and carted eight miles during the hottest part of the hottest weather. From this milk the writer, Dr. Newton, was able to separate the poisonous substance, tryotoxicon, first discovered by Prof. Vaughan, of Michigan University, in poisonous cheese. This is probably a ptomaine, and forms distinct needle-shaped crystals, which give a blue color with a mixture of potassium ferrocyanide and ferric chloride and reduced iodic acid. Placed on the tongue the crystals give a burning sensation. Fed to a cat they produce retching and vomiting and collapse.

It would seem as if we had here a perfect demonstration that milk under certain circumstances will undergo such changes as to become poisonous.

Careful dairymen cool their milk in open vessels before placing it in cans for delivery at the market.—*American Lancet.*

**Investigations on the Action of Micrococci of Vaccine.**—Dr. L. Voight has presented an interesting study of vaccine cultures, an abstract of which follows:

Koch and Feiler, as the result of their researches, conclude that the micro-organisms which are regularly found in vaccine lymph are pathogenic but do not contain the vaccine contagium, and that when isolated and used for vaccination neither produce a pock nor grant protection.

Quist, of Helsingpors, has grown vaccine lymph in various media and successfully vaccinated children with the products.

Bareggi has stated that the micro-organisms of small-pox and vaccine are identical. He has described large and small cocci, which latter are probably the carriers of the contagion. They exhibit active Brownian movements, and develop so rapidly that in three days a kilogramme of artificial lymph may be prepared from a capillary tube of human lymph. With such cultures, six out of eight vaccinations were successful.

The author has separated and cultivated in gelatin three kinds of organisms which all liquefy the medium.

1. Small cocci which, near the surface of the gelatin, form grayish-white, sharply defined spherical colonies, the periphery being of pale yellow color. Under a high power they appear to possess a proper movement, and occur in knots of two and four. These are probably the carriers of contagion.

2. Large cocci which form pale green spherical colonies with rough and indefinite surface, and under a high power show only slight Brownian movement. These were not always present.

3. Small cocci in gray, yellow, dark, sharply defined colonies with a delicate cloud in the neighborhood, presenting under a high power groups of two or four, but possessing no spontaneous movement. In stained preparations these are indistinguishable from (1), but are ineffective for vaccination.

Three out of fourteen calves were successfully vaccinated with (1).

The author offers his results as a partial contribution to the subject, which requires further investigation.—*Manchester Med. Chronicle.*

**Note on the Treatment of Thread-Worms in Children.**—The complete cure of thread-worms in children is often very difficult. While the ordinary methods used, such as rectal injections of salt and water, infusion of quassia, and other remedies, do good for a time, yet they often fail to relieve the attendant symptoms of "worms," symptoms usually very irregular, and in some cases severe, in character. In many cases, though the irritation about the anus is relieved by injections, the irregularity of the bowels and the disturbance of sleep remain the same. This is probably due to the fact that the habitat of the worms is higher up in the large intestine, where no remedy introduced by the rectum can reach them.

In many cases I have found that rhubarb in small doses brings away large numbers of worms, and at the same time regulates the bowels, so that the use of injections may in most cases be dispensed with. The formula which I have found most useful is as follows, varying slightly with the age of the child:

℞. Tincture rhei.............................*m* iij.

Magnesii carbonatis........................gr. iij.

Tincturæ zingiberis.........................*m* j.

Aquam, ad...............................ʒ j.

This is to be taken twice or three times daily according to the effect on the bowels. Whether the rhubarb acts as a vermicide or simply by "moving the worms on," I am unable to say.—*The Practitioner.*

**Vertigo.**—Dr. Willard (in *New York Med. Record*) found the following the best treatment in obstinate cases of vertigo:

After trying the various remedies recommended in the textbooks, and not deriving the results I desired, I applied blisters to the neck, and in some cases, when the pain in the back was severe, all the way down the spine. The result of the blistering was very satisfactory, improvement beginning almost immediately, and my patients are to-day attending to their ordinary duties. Of course I did not neglect, but continued general treatment. I think that the use of blisters in these cases cannot be too highly recommended, even though we cannot explain the *modus operandi.* In the treatment of these cases I used a blister which does not contain cantharides, and which is free from the exceedingly unpleasant complications of strangury. These blisters, or "issue plasters" as they are called, have proved very satisfactory in my hands, not once producing unpleasant symptoms — they blister quickly, without pain of any consequence, and by returning them to the blistered surface an issue can be kept up for any desirable length of time — and, as I remarked above, without the slightest fear of straugury occurring as a complication. I greatly fear that with the general practitioner the beneficial effects of judicious blistering in these and other similar cases, is oftentimes lost sight of in dread of the unpleasant symptoms produced by cantharides.

**Stone in the Bladder — Its Surgical Treatment.**— Dr. William Cadge (*British Med. Journal*) in an interesting series of lectures on this subject summarizes his views thus:

1. In children litholapaxy should be more frequently adopted than has been the custom.

2. In male children, when the stone is at all large, the suprapubic will probably prove easier to do, and safer than the perineal operation.

3. In female children lithrolapaxy should be the rule for small stones, and the high operation for all large ones.

4. In adult females litholapaxy, or dilatation and extraction, should be adopted for stones of moderate size ; vaginal lithotcmy for those somewhat larger ; and the high operation for stones of decidedly large size.

5. In adult males litholapaxy should be the rule for stones up to an ounce, or an ounce and a half ; above that size, lateral or possibly suprapubic lithotomy — certainly the latter for all stones over three ounces.

6. In the aged the same rules apply when the urinary organs are healthy, but when the prostate is large and the bladder atonic, suprapubic lithotomy should be adopted, until its success or failure is demonstrated —*Amer. Lancet.*

**Typhoid Fever,** as always, is reported to be most prevalent in the imperfectly sewered cities. Deaths reported from it for the three months ending September 30th, in Paris, 279 ; Lyons, 58 ; Warsaw, 58 ; St. Petersburg, 159 ; Alexandria, 51 ; Cairo, 226. But even in these the mortality from this cause is less in proportion than in some American cities equally, if not, indeed, more neglectful of efficient sewerage. Philadelphia and Baltimore are prominent examples ; in the former, with bad sewerage, for the same period, the mortality from typhoid fever was 101 ; in the latter, without sewerage, about the same proportion to population, 57. Very few cities of much magnitude abroad, as at home, are wholly exempt from typhoid fever, but proportionally those are most so which are best sewered and drained. Deaths reported by 14 large English cities, comprising about 8,500,000 population, for months ending September 30, was only 438—173 (4,149,533 inhabitants) in London. In Berlin, 1315,287 inhabitants, 75. In Munich, which until recent sewage was one of the most fruitful sources of typhoid fever of any city in Europe, 19 ; Brussels, 8.—*The Sanitarian.*

**Podophyllum Hypodermically.**— Brunton states that the resin of podophyllum "acts on the bowels when injected subcutaneously, as well as when introduced into the intestinal canal." In 1880, Dr. D. O. Brau reported that he had used podophyllotoxin as a purgative for children and found it completely to represent podophyllin. To a child ten year old he gave from 6-100 to 9-100 of a grain.

We have used podophyllotoxin upon kittens three times, producing free catharsis in each case within an hour. In two of the experiments catharsis was carried to the extent of bloody, mucous passages, the bowels remaining loose for several days. In these cases there was no depression of any kind manifest until after many watery stools had been voided, when the cats betrayed the weakening effect of this drain of albuminous fluid. In one case the catharsis was promptly checked by the administration of 1-16 of a grain of morphine. We have also used podophyllotoxin upon man four times — two of the cases being in the Cincinnati Hospital.—*Med. News.*

**Laparotomy as an Aid to Herniotomy.**— Fenwick records a case in the September *London Lancet* (1885), showing the value of laparotomy as an aid to herniotomy. The operation owes its origin to a suggestion of Mr. Annandale, who pointed out how much easier it is to draw a loop of bowel up than to press it back, piece by piece, into the peritoneal cavity. A man, aged fifty-three, was admitted into the London Hospital with a strangulated left inguinal hernia. All attempts at reduction failed, and an operation was decided upon.

The sac was opened and the neck incised, but every time a piece of the gut was pushed back into the peritoneal cavity it shot out again. The author then decided to open the abdominal cavity, and made a two-inch incision just above the pubes. The left forefinger was introduced into the abdomen and hooked under the gut, and the entire gut was easily drawn into the abdomen. The patient, however, died six days after the operation from exhaustion. The author advocates this method of treatment in cases of strangulated hernia, and believes that the small abdominal wound would not add to the risk of herniotomy.

**She Was Not Spayed.**— The *Medical Press and Circular*, September 8, 1886, vouches for the truth of the following:

"A certain operator proposed to remove 'the uterine appendages' from a patient under his care, and he asked a gentleman present if he would like to examine the patient, saying she had been suffering for years with an acute pain, that life was a burden, etc. The gentleman, struck by the full, round, rosy cheeks and red lips of the woman, first asked her how she slept nights. 'Oh, very well, thank you,' was the reply. 'You have no pain at night, then?' was the next question. 'Oh, no,' she answered. 'Have you pain all day long?' continued the questioner. 'Not all the day through,' she replied. 'Have you pain every day?' he went on. 'No, not every day,' was the answer. 'How often does the pain come on then?' the gentleman continued. 'Oh, three or four days a month,' was the innocent reply, much to the amazement of the questioner. The would-be operator was heard to mutter something about 'getting any answer you like,' but it will be satisfactory to learn that the patient saved her ovaries on that occasion, at least."—*Med. and Surg. Reporter.*

**Treatment of Pruritus Pudendi.**—In a paper read before the Cincinnati Academy of Medicine, September 20, 1886, on Pruritus Pudenti, the author (Dr. E. S. McKee) discussed that interesting section, the treatment, as follows:

First, we should ascertain the cause of the disease to treat it intelligently. We should treat the constitutional diseases as the origin of the trouble. Next, we should treat the morbid phenomena, the pruritus. Remove the cause, and the pruritus will disappear of itself. The parts should be washed twice a day with castile soap and water. The diet should be vegetable, and regular action of the bowels maintained. As a general rule, stimulants should be disallowed.

In this troublesome trouble, for we can hardly call it a disease, we need all the remedies we can find; hence I give all I know.

4-per cent. solution of boracic acid.
3-10-per cent. solution of carbolic acid.
3-5-per cent. solution of argenti nitratis.
0.5-per cent. solution of bichloride of mercury.
25-50 per cent. solution of sulphurous acid.
6-per cent. solution of sodii biborat.

Ointments of tar, boracic acid, camphor, or iodoform, mixtures of camphor and chloral, infusion of tobacco, 20-per cent. solution of chloroform in almond oil.

Treatment with the bichloride should be preceded by a removal of the mucus with warm water, and then dry with soft linen. Pass a sponge, moistened with the solution, rapidly over the affected part. This leaves a smarting, burning sensation, which is alleviated by a few minutes' washing with cold water. Subsequent applications become less and less painful.

M. Dubois recommends, in the rebellious cases, that the entire surface of the vulva be cauterized with a solid stick of the nitrate of silver. The great objection to this is that it is extremely painful, and the alleviation produced by it is almost always temporary. Meigs recommends:

℞. Borax .......................... ʒ ij,

Morph. sulph .......................... gr. ivss,

Aquæ rosæ dest .......................... ℥ viij.

M. S. Apply three times a day to the affected part with a sponge or soft piece of linen. Take care to wash well the parts beforehand with soap and water, and dry them well afterward. A compress dipped in the oil of sweet almonds and laid in the commissure of the vagina is recommended.

When the trouble is general, temporary relief may be obtained by placing the woman in a prolonged soda bath, and subsequently rubbing the entire surface with vasoline.

Pruritus which has extended upon the distended abdominal walls is well treated with—

℞. Lin. saponis comp. . . . . . . . . . . . . . . . . . . . . . . . . . . . . . ℥ v,
Chloroform . . . . . . . . . . . . . . . . . . . . . . . . . . . . . . . . . . . ℥ j.

S. Apply locally.

If the itching comes from an ulcerated cervix, or, more properly, from the irritating discharge proceeding from it, apply nitrate of silver, and introduce a tampon of tanno-glycerine.

Pruritus from breeding pediculi is well treated by mild mercurial ointments. Stavesacre answers well. A plasma formed of flour of sulphur and water, saline purgatives, as Pullna or Friedichshall water. Vichy baths, or even bathing with cold or tepid water, constitute the best polliatives. Salines and colchicum may be indicated, also bromide of potassium. A weak solution of Goulard's lotion, or a lotion composed of—

℞. Liq. morphiæ hydrochlorate. . . . . . . . . . . . . . . . . . . . . ℥ j,

Acid hydrocyanic . . . . . . . . . . . . . . . . . . . . . . . . . . . ʒ iss,

Avuæ. . . . . . . . . . . . . . . . . . . . . . . . . . . . . . . . . . . ℥ vj.

M. S. Use as a lotion

Pledgets soaked in the following and placed in the vagina have been found useful :

℞. Acidi sulphuric,

Sodii biborate,

Acidi sulphurici,

Glycerini,

} aa. . . . . . . . . . . . . . . . . . . . . . . . . . . ʒ ij.

M. Insert at bedtime and withdraw in the morning.

Iodoform may be dusted over the parts. The following has often given relief :

℞. Chloroformi . . . . . . . . . . . . . . . . . . . . . . . . . . . . . . ʒ ij,

Ol. amygdal. . . . . . . . . . . . . . . . . . . . . . . . . . . . . . . . ℥ ij.

M. S. Apply externally.

Morphia and chloral internally may be found necessary to obtain relief at night. Hildebrant has found the tincture cannabis indicæ, 10–20 gtts., to be of even more benefit than these.—*Med. & Surg. Reporter.*

**Cholera.**—During the three months ending September 30, deaths reported in Buda Pesth, 169 ; during the four weeks ending October 23, 303, the number of cases not reported. In Trieste, during the three months ending September 30, 411 deaths ; during the four weeks ending October 23, 110. In Venice, during the three months ending September 30, 132 deaths. In Bologna, during the month of September, 67 deaths. In Szegedin, during three weeks ending October 19, 64 deaths. In Tagliari, October 6–9, 13 deaths. In Tarentum, October 6–10, 4 deaths. (Telegraphic :) Buenos Ayres, November 19 (via Galveston). Cholera has broken out in the hospital for the insane in this city, and of 18 persons attacked 2 have died. There were 8 new cases and 5 deaths at Rosario during the past twenty-four hours.

**Incontinence of Urine.**—Owing to the care needed in the administration of strychnia, some practitioners are now making successful use of ergot in these cases. Like strychnia, it certainly has the power of causing muscular contraction.

In certain cases where the augmentation of the vesical contractility appears to be associated with a weakness of the urethral muscles, strychnia or ergot—which would here be better may be used in conjunction with the belladonna.

Of all the remedies used for incontinence caused by contractile insufficiency in the urethral muscles, electricity is, perhaps, the most efficacious. One of the poles may be applied over the perineum and the other upon the abdomen over the bladder, or in the rectum.

Grusse reports many cures by this method. In obstinate cases one of the poles introduced into the urethra and the other applied to the hypogastrium, the perineum, or is introduced into the rectum. The use of this method sometimes frightens children and their parents, but it is not painful. Its effect, when it cures, is almost immediate, and when it does not cure it affords great relief. The peptonate of iron is a proper adjuvant to use simultaneously with ergot, strychnia or electricity, or if these give special tone to the muscular fibre, the albuminate fortifies the general system and reconstitutes the blood corpuscles. Hydropathy, like iron, is a powerful tenic, but should be used prudently. Sea baths are often successful with lymphatic and scrofulous subjects, and sulphur baths in cases of nervous affections.

Incontinent children should take their evening meal early and be somewhat restricted in the use of drinking water at night.

Parents should be directed to find out the hour at which the child is likely to urinate in bed, and wake him up a short time before it arrives. In the daytime care should be taken to encourage him to urinate only at certain hours, fixed at a reasonable time apart. This is the best way to habituate the bladder to retaining urine. Where it is certain that incontinence is caused by carelessness, prudent correction should be administered. Trousseau tells of an obstinate case of incontinence in a full-grown girl, whose malady had resisted all medicaments, but gave way at last to the nightly attacks of a determined mother armed with a whip.—DR. H. PICARD, *in Chicago Med. Times.*

**The Water Diet.**—Dr. Almanera Butler, of Chili, S. A., advocates what he calls the Water Diet in the treatment of cholera infantum. One of the most prominent symptoms of the disease being intense thirst, it was taken for granted that Nature pointed out the required treatment. Pure cold water is given in quantities as large as the child's desires. Dr. Luton, of Rheims, was the first to advocate this treatment. The following are the principles advocated by him :

1. Stop all food, for this is the probable cause of the disease.
2. Give pure cold water in unlimited quantities. This acts as a tonic to the intestines and increases the volume of blood.
3. Gradually return to a nutritious diet. Starches and sugar should be prohibited until patient has recovered. This treatment will not interfere with the usual pharmacological remedies.

Dr. Luton advocates the hypodermic use of morphine, and nitrate of silver internally.—*Buffalo Med. & Surg. Journal.*

**Potassium Permanganate in Amenorrhœa.**—Dr. Marshall, of San Francisco, after employing this drug in fifty cases of amenorrhœa, has arrived at the following conclusions:

1. The permanganate acts satisfactorily in about 70 per cent of the "selected cases."

2. It should be administered one or two hours *after* eating.

The disagreeable action on the stomach may be relieved by combining it with the following:

| | |
|---|---|
| Oxalate of cerium | 1 grain. |
| Hydrochloride of cocaine | 1-6 grain. |
| Subnitrate bismuth | 5 grain. |
| Powdered ipecac | 1-60 grain. |

The writer also states that this drug has a marked tonic effect, and generally causes mental exhilaration.—*Canadian Practitioner.*

**Antiseptic Treatment of Carbuncle.**—This is suggested by the recent discovery that the disease is due to the evolution of a microbe. When the lesions are already deep, or if subcutaneous abscesses have formed beneath the cite of the carbuncle, surgical interference becomes of course necessary in order to attain complete disinfection; but when the parasite has not yet penetrated or invaded the deeper tissues, its further progress may be arrested by timely antiseptic treatment. A solution of corrosive sublimate, 1 in 1000, may be sprayed into the morbid surface, whilst gentle pressure is employed around the carbuncle, to bring to the surface any pus that may be there. Afterwards a paste of boric acid may be applied over the whole diseased surface, held *in situ* by salicylic cotton and antiseptic band. This treatment is especially applicable to carbuncle of the lip, a not uncommon malady; the pain and tension rapidly subside and the patient will convalesce in less than two weeks, in an ordinary case.—*Medical World.*

**An Incompatible of Antipyrine.**—In the *New York Medical Journal*, October 24, 1885, Dr. Eccles announces that while making pharmaceutical investigations he had occasion to mix some sweet spirits of nitre with a solution of antipyrine. At first the mixture was clear and colorless, but after some hours of standing became green. The optical appearances of this fluid were identical with those of the anilines, and, as antipyrine was a coal-tar product, he supposed the change was due to the formation of a green aniline, and so it had proved to be. This incompatibility was all the more important as both substances were used for antipyretic purposes, and might be prescribed together.

**An Item.**—Dr. W. C. Maxey, of Marcus, Io., writes us as follows: "You may say to your readers that when the lines of graduation on clinical thermometers, graduated glasses, urinometers, etc., become dim and illegible from use and wear, that a little writing ink, rubbed over the marked surface, will surprise them at the prominence and clearness with which the lines and figures are again brought into view.—*Practitioner.*

**Ingluvin.**—It has been a favorite saying among the more distinguished of our profession, that there are a few essential drugs without which the practice of medicine would be impossible, and that when we have selected these few, the great multitude of articles in our materia medica are comparatively useless. This is a very true idea. With calomel, opium, castor oil, quinine, mercury, a few such standard drugs, the physician is usually equipped to meet all emergencies. Almost weekly some new drug is brought to our notice, but in many instances, after trial, it is found either inferior to, or no better than, those which we already have, and its use is dispensed with. But it does sometimes happen that we are offered an article of such undoubted merit that it is warranted in taking rank with the standard articles of our materia medica. Such an article is ingluvin. (Ingluvin is a refined substance prepared from the ventriculosus callosus gallinaceus, the gizzard of the domestic fowl, gans domesticus.) It is the essential principle of the gizzard, and bears the same relation to poultry that pepsin does to the higher animals.

A favorite prescription of Chinese physicians for chronic indigestion is to cut up and digest chicken gizzards in hot water until they are reduced to a pulp, and then add some spices. A tablespoonful or two of the resulting paste is taken at each meal until the patient has entirely recovered. From China the practice passed to other parts of Asia, and was adopted here and there among the Mediterranean peoples. Strange to say, it was never learned by the great nations of Europe until the latter part of the present century.

The diseases in which the use of ingluvin is indicated are indigestion in its various forms, known as dyspepsia, and for sick stomach or nausea caused by debility of that organ. It was originally discovered to be a remedy, indeed a specific, for vomiting in pregnancy; in this respect it stands above all other medicinal agents. In all that is here set forth the manufacturers claim no more than is sustained by medical authority of the highest standard.

In ingluvin, the physician has what might be called a specific for a sickness which in many cases has hitherto been uncontrollable.

Ingluvin is a powder of a yellowish-gray color, and may be prescribed in the same manner, dose, and combinations as pepsin, 3 to 10 grs. The pulverulent form is considered more desirable, and it can be administered either dry or in water, milk, or tea. In sickness in gestation, the dose may be increased to 10 or 20 grains.

The following will make a nice formula in which to prescribe it for the vomiting of pregnancy. It was thus used successfully by Dr. George F. Meeser, of this city:

℞. Ingluvin......................................ʒ j,

Bismuth subnit...................................ʒ ss.

Sig.—One every three hours.

Oxalate of cerium may be prescribed with it, 1 to 3 grains to each dose.

Dr. Shelly recommends the following formulæ for diarrhœa, cholera infantum, and marasmus:

INFANT FORMULA.

℞. Ingluvin......................................gr. xij,
Sacch. lac.......................................gr. x.

Misce et ft, cht. No. x.

Sig.—One every four hours.

℞. Aquæ calcis...................................f ℥ ij,

Spts. lavland comp., } aa........................f ʒ j,

Syr. rhei arom., }

Tr. opii.........................................gtt. x.

Misce. Sig.—A teaspoonful every two to four hours.

FOR ADULTS.

℞. Ingluvin......................................f ʒ j,

Morphiæ sulph....................................gr. jss.

Misce et ft. cht. No. xii.
Sig.—One every four to six hours.

℞. Aquæ calcis...................................f ℥ ijss,

Spts. lavand. comp...............................f ℥ ss,

Syr. rhei arom...................................f ʒ vj,

Tr. opii.........................................f ʒ ss.

Misce Sig.—Dessertspoonful every two to four hours, or after each evacuation.

The substance ingluvin without any combination has also yielded almost constantly satisfactory results.

Dr. Roberts Bartholow, who probably stands to-day as the greatest authority on materia medica in this country, speaking of ingluvin says:

"Ingluvin has the remarkable property of arresting certain kinds of vomiting—notably the *vomiting of pregnancy*. It is a stomachic tonic, and relieves *indigestion*, *flatulence* and *dyspepsia*.

"The author's experience is confirmatory of the statements which have been put forth regarding the exceptional power of this agent to arrest the vomiting of pregnancy. It can be administered in inflammatory conditions of the mucous membrane, as it has no irritant effect. Under ordinary circumstances, and when the object of its administration is to promote the digestive function, it should be administered after meals. When the object is to arrest the vomiting of pregnancy, it should be given before meals."

**Surgical Myxœdema.**—Total ablation of the thyroid gland is liable to be followed by a remarkable constitutional affection called surgical or operative myxœdema, in contradistinction to the disease of the same nature which occurs spontaneously. The surgeons in certain of the Swiss cantons in which bronchocele is endemic have had a large experience with this remarkable condition, and some months ago we referred to the conclusions of Kocher as well as to the artificial production of the disease in monkeys by Horsley.

At the recent Surgical congress at Paris, Reverdin, of Geneva, presented a paper on the identity of the operative and spontaneous myxœdema. The earliest and most important of the symptoms of operative myxœdema is swelling of the face, hands, and neck ; not the puffy œdema of dropsy, but a firmer, more solid exudation. The tactile sensation is slow, the special senses are dulled, and there are often unpleasant subjective sensations, particularly that of cold. The skin becomes dry and scaly, and the hair often falls ; speech becomes slow, and there is loss of memory. With these, there are no visceral symptoms, though occasionally the urine is albuminous. In children from whom the gland has been removed, a condition of cretinism may be induced and there may be such an arrest of the physical and mental development that the child becomes an idiot. Bourneville and Bricon have observed myxœdema in idiotic children, and have determined the absence of the thyroid gland in these cases.

It is well to know that this condition does not invariably follow removal of the gland. Thus, of Kocher's thirty cases twenty-four became myxœdematous ; of Reverdin's eleven, only five. On the other hand, Billroth, in a large number of operations, has not had a case, and Bottini has been equally fortunate. It would certainly seem as if, in Switzerland, where goitre is endemic in certain regions, there are other conditions which render the patients more liable to this unfortunate event. The extirpation need not be total; Reverdin has seen a mild grade of the disease follow removal of only one lobe. There are many different grades of the affection and the milder forms are capable of amelioration and, occasionally, of cure.—*Medical News.*

**For Gleet and Chronic Gonorrhœa.**—Dr. O. D. Ball (in *Albany Medical Annals*) recommends warmly the following :

| | |
|---|---|
| Zinc oxide | 3 drachms, |
| Lard | 3 drachms, |
| Simple cerate | 2 drachm. |

The method of using this ointment, which will adhere nicely to the bougie and yet be soft enough to apply itself to the urethral membrane, is to fill the constricted portion of the bougie out evenly and as smoothly as possible with the full calibre of the instrument, which may be lubricated with vaseline if the parts are sensitive The bougie should be carried at once to the prostatic portion of the urethra as rapidly as possible, and then, after rotating it in both directions, it should be slightly withdrawn and then pushed back again, treating the remaining portion of the urethra in the same way. The patient should always empty his bladder previous to the application, and should be instructed to refrain from doing so again as long as possible. The application should be made at least twice a day.

**A New Hæmostatic.**—Dr. Spaak, in the *Journal de Bruxelles*, describes a hæmostatic which he accidentally discovered, and which he has used for some months. It consists of two parts of chloroform and a hundred parts water, and presents the following advantages:

1. It acts with remarkable promptness.
2. It has not the least unpleasant taste.
3. It has no escharotic action.
4. It is always to be had, and costs almost nothing.
5. It has no unpleasantness in its action, and does not disturb the operation.

In all operations in the cavity of the mouth and neck, a simple washing out with this remedy is sufficient to stop the hemorrhage from the larger vessels in an instant.

The author does not state the reason of this action; he simply relates the fact.—*Nash. Jour. of Med. and Surg.*

**Ophthalmia Neonatorum.**—J. E. Weeks, M. D., gives in the *Medical Record* of July 24, 1886, the treatment of this disease as followed at the New York Ophthalmic and Aural Institute:

The conjunctival sacs are cleansed thoroughly every half hour, or more often if the discharge is profuse; constant applications of cold to the lids by the use of small pieces of linen, which are placed on a cake of ice near the patient, and are changed to the eyes every half minute, or as soon as the piece on the lid becomes warm another piece is substituted. The conjunctivæ are brushed with a one or two per cent. solution of nitrate of silver morning and night, commencing when the discharge becomes profuse. If corneal complications arise, one or two drops of a one-half per cent. solution of atropine are instilled twice or three times a day. —*American Lancet.*

**Gelosin** is a mucilaginous substance extracted from Gelidium corneum (*Lamouroux*), an alga of Japan, and is found in commerce as dry, whitish fragments, extremely flexible. Gelosin forms an excellent vehicle for the administration of soluble medicaments or for making suppositories, cataplasms, bougies, etc. M. Guerin has presented to the Societe de Therapeutique, of Paris, some specimens of gelosin medicated with camphor, creasote, sulphate of zinc, turpeth mineral, cocaine, extract of belladonna, iodoform, corrosive sublimate, carbolic acid, coal tar, etc. To manipulate this substance all that is required is to add an equal weight of warm water to dissolve it, and then to incorporate with it the medicament. Conveniently sterilized gelosin might be advantageously employed in bacteriological researches.—*Jour. de Phar. et. de Chimie.*

**Substitute for Fehling's Solution.**—Professor Holland gives the following as a test for sugar; it is very efficient, easily prepared, and is not spoiled by keeping:

Cupric sulphate.................................ʒ i.

Glycerine.......................................℥ i.

To make the test, add 5 drops of this solution to 1 drachm of liquor potasse in a test tube. Boil a few minutes to test the purity of the fluid. Should it remain clear, then add a few drops of urine. If glucose be present in quantity there is at once thrown down a red precipitate, just as in the ordinary Fehling test. To detect minute amounts of sugar, not shown by the above procedure, after making the test as above, add ½ a drachm of urine; boil and set aside. If the sugar be present even in very minute quantities, the liquid, as it cools, will turn of an olive-green color and become turbid.—*Canadian Practitioner.*

**Lister's Latest Antiseptic Dressing.**—Lister's latest antiseptic dressing is known as salalembroth. He uses it exclusively in his wards, with fine results. It is a double mercurial salt, made by the sublimation of a mixture of perchloride of mercury and chloride of ammonium. It is very soluble, and has not been used in medicine since the time of the alchemists. All dressings—gauze, cotton, wool, bandages, lint, bedding, patient's undercloth-ing, etc.—are soaked in a 1 to 100 solution, and dried. He colors these dressings with analine blue, 1 to 10,000, so that when an alkaline discharge comes in contact with the dressings, the blue is removed and turns reddish, enabling him to see where the discharge has been and its quantity, however small or large, moist or dried.—*Med. and Surg. Reporter.*

**Hemirheumatism.**—At a recent meeting of the Academy of Medicine of Paris, there was presented, for Dr. Cazalis, a note on the hemilateral predominance of the manifestations of chronic rheumatism. According to the author, a tendency to unilateral rheumatism is noted in two-thirds of such cases, the right side being most frequently affected. When pulmonary congestion, or bronchitis of arthritic nature appear in these patients, they are most often located on the side on which the rheumatism predominates. On this side also are most frequently found deviations and deformity of the great toe. If hemicrania be added to the various modalities of arthritism, there will be found still greater ground for admitting that the central system exerts a great influence in the localization of chronic manifestations.—*Medical News.*

**Ingrowing Toe-nail.**—For ingrowing toe-nail, M. Lucas-Championniere recommends washing the toe with a 5-per cent. solution of carbolic acid. Local anæsthesia is produced, the nail is removed by avulsion, and the rim of the bed of the nail is excised. Carbolic acid is again used, and a salicylic acid cotton-wool dressing impregnated with vaseline, containing a fifth of boric acid, is applied; it is then powdered over with iodoform, and enveloped in salicylic cotton-wool, which is again enveloped in ordinary cotton-wool, and finally bandaged. After an interval of three weeks there is cicatrization, and the dressing is removed.—*Medical and Surgical Reporter.*

**Calomel in Diphtheria.**—Dr. W. H. Daly thus summarizes his treatment of diphtheria:

1. Give calomel in its purity.
2. Give it in large doses.
3. Give it frequently.
4. Give it until you have the free and characteristic catharsis.
5. Give light, nutritious diet.
6. Give little or no other medicine.—*N. Y. Med. Journal.*

**Hepatic Colic.**—Prof. Bartholow had at his clinic a patient with *hepatic colic*, who was not jaundiced. The stone may be of such a size that suffering is produced by its passage through the cystic duct, while it passes without pain through the common duct, and without obstruction; therefore jaundice is not produced. To keep the bile alkaline and so prevent the further formation of gall stones, give persistently sodium phosphate or cholate.—*American Medical Digest.*

Dr. Neff, at a recent clinic, formulated the following comparatively simple treatment for *acute pleurisy.* Strap the affected side of the chest firmly with adhesive strips, having previously used dry cups over the part; thus you procure rest. Give pulvis ipecac. et opii, in gr. iv doses, every four hours, for quiet and sleep; if more opiate be required, use morphine hypodermically.—*Ibid.*

**Treatment of uncontrollable vomiting** of pregnant women. Dujardin-Beaumetz gives the following: ℞. Cocaine hydrochloratis, 0.50; aquæ destillate, 300 (8⅓ to 10 ounces of water). Dose, two tablespoonfuls every hour. The patient should lie down to prevent vertigo.—*Jour. de Phar. et. de Chimie.*

**An etheral solution of tannin,** of syrupy consistence, is said to be the best application to burns. It immediately soothes the intense pain, dries rapidly and forms a pliable, non-elastic coating which is preferable to collodion, because it does not shrink and become stiff.—*Ex.*

**Albuminuria**—Dr. Broadbent says: "In the numerous cases of albuminuria in the London Fever Hospital, towards the decline of the affection, when only small quantities of albumen remain, mercury in limited doses usually leads to the entire disappearance of the albumen."

**Barbasco—A New Mexican Drug.**—The *Pharmaceutical Record*, November, 1886, reports the importation from Mexico of specimens of this new drug, which has been found useful in removing hair in skin diseases.

